# Parental Serotonin Modulation Alters Monoamine Balance in Identified Neurons and Affects Locomotor Activity in Progeny of *Lymnaea stagnalis* (Mollusca: Gastropoda)

**DOI:** 10.3390/ijms26062454

**Published:** 2025-03-10

**Authors:** Anastasiia Shestipalova, Viktoriya Nikishchenko, Anton Bogomolov, Elena E. Voronezhskaya

**Affiliations:** 1 Koltsov Institute of Developmental Biology, Russian Academy of Sciences, Moscow 119334, Russia; anastasia472233@gmail.com (A.S.); bogomolov.anton2000@gmail.com (A.B.); 2 A.V. Zhirmunsky National Scientific Center of Marine Biology, Far Eastern Branch, Russian Academy of Sciences, Vladivostok 690041, Russia; niktori2000@gmail.com

**Keywords:** apical sensory organ, embryonic locomotion, seasonal serotonin alteration, maternal serotonin, identified monoaminergic neuron, gastropod mollusk

## Abstract

Monoamine neurotransmitters play a critical role in the development and function of the nervous system. In this study, we investigated the impact of parental serotonin (5-HT) modulation on the monoamine balance in the identified apical neurons of *Lymnaea stagnalis* embryos and its influence on embryonic locomotor activity. Using immunocytochemical and pharmacological approaches, we detected serotonin in the apical neurons of veliger-stage embryos, observing that the relative 5-HT level within these neurons varied with seasonal conditions. Pharmacological elevation of parental 5-HT levels significantly increased the relative 5-HT level in the oocytes and subsequently in the apical neurons of their offspring. Notably, while the relative dopamine (DA) levels in these neurons remained stable, the increase in the relative 5-HT level significantly enhanced the embryos’ rotational locomotion. The expression of tryptophan hydroxylase (*TPH*), a key enzyme in serotonin synthesis, is a prerequisite for the elevation of the relative 5-HT level in apical neurons and is detected as early as the gastrula stage. Importantly, neither a reduction of 5-HT in the maternal organism by chlorpromazine application nor its pharmacological elevation via serotonin precursor (5-HTP) application at the cleavage stage affected the monoamine balance in apical neurons. These findings provide novel insights into how the parental 5-HT level selectively alters the monoamine phenotype of the identified neurons, offering a model for studying environmentally induced neural plasticity in early development.

## 1. Introduction

Each neuron in an adult organism, whether vertebrate or invertebrate, possesses a specific phenotype. This neuronal phenotype includes, among other characteristics, neurotransmitter specificity—the ability to synthesize particular substances used for cell-to-cell communication and interaction with effectors. The distinctive neuronal phenotype is established during cell differentiation. For a long time, it was assumed that each neuron could synthesize only one type of neurotransmitter (Dale’s principle of “one neuron–one neurotransmitter”). However, it soon became evident that a single neuron could produce multiple neurotransmitters [1]. Moreover, some of these neurotransmitters serve as primary messengers, directly involved in signal transmission, while others act as modulators, influencing the release and effects of the primary neurotransmitter. Among the well-known primary transmitters are the biogenic amines serotonin (5-HT) and dopamine (DA), which are typically expressed in distinct neurons and neuronal populations. The remarkable coexistence of both dopamine and serotonin within a single identified neuron has been reported in the lobster nervous system [2], and serotonin, histamine, and octopamine in identified neurons of the mollusk Aplysia [3]. The authors emphasize the significance of their findings, as a neuron with dual neurotransmitter expression may play a unique role in modulating complex behaviors and have a broader role in the integrative processing of information than previously thought.

Co-expression of neurotransmitters such as serotonin and dopamine in single neurons encourages the hypothesis of its potential evolutionary significance. In terms of neurobiology, the spatial and temporal regulation of these two primary neurotransmitters within individual neurons could provide an adaptive advantage by enhancing the flexibility of neuronal responses to environmental stimuli. For example, both serotonin and dopamine are involved in regulating mood, motor control, and decision-making processes [4,5,6]. A neuron co-releasing these transmitters could fine-tune its output, offering a more dynamic and context-dependent modulation of behavior.

From an evolutionary perspective, the presence of multiple neurotransmitters within a single neuron could represent an efficient strategy for the nervous system to manage complex behaviors without requiring a larger number of distinct neuronal populations. This is particularly important in the case of the larval nervous system, which contains a limited number of neurons. Neurotransmitter duality may also reflect an evolutionary adaptation to reduce the metabolic cost of maintaining the multiple machinery of neurotransmitter synthesis. In the case of 5-HT and DA, only the first-step synthesis enzymes are different—tyrosine hydroxylase and tryptophan hydroxylase—while the last-step synthesis enzyme is similar: aromatic amino acid decarboxylase.

Investigation of the differentiation pathway of dual neurotransmitter neurons during embryogenesis is essential for understanding how such cells contribute to complex neural circuits, optimize neural efficiency, and regulate behavior while also providing insights into evolutionary and environmental adaptations and potential therapeutic targets for neurological disorders. This task is particularly challenging in vertebrate and higher invertebrate nervous systems due to their complex neural architecture and regulatory mechanisms. Therefore, alternative developmental models should be utilized to facilitate the investigation of how neurons acquire their final phenotype during development and the potential plasticity of this process.

Gastropod mollusks serve as a convenient model for studying the differentiation of individual neurons, neuronal genealogy, and neuronal origin in evolution [7,8]. Mollusks are characterized by a highly determined neuronal phenotype, where neurons with identical specificity can be found in the same locations within the nervous systems of mollusks across different groups [7,9,10,11]. Additionally, during development, mollusks exhibit a characteristic feature: a neuronal complex known as the apical organ. The apical organ (AO), often referred to as the “larval brain”, represents the primary sensory structure in the larvae of many invertebrates. Signals from AO neurons mediate complex embryonic responses, regulating developmental rates, locomotor activity, and the onset of metamorphosis [8]. A characteristic feature of the AO is the presence of sensory apical neurons that contain the monoamine serotonin (5-HT) [12]. However, in some cases, apical neurons have also been found to contain the catecholamine dopamine (DA) [8].

This scenario is observed during the development of the freshwater mollusk *Lymnaea stagnalis*. The apical neurons of *L. stagnalis* predominantly contain dopamine (DA), with serotonin (5-HT) being present in these cells only occasionally [13]. Notably, during an extended developmental period—from the trochophore to the mid-veliger stage—DA in freshwater snail embryos is exclusively found in the neurons of the apical organ. Other DA containing cells in the ganglia of the central nervous system appear only after the embryo undergoes metamorphosis [8,14,15,16]. It has been shown that DA and 5-HT, secreted by the apical neurons of the freshwater gastropods *L. stagnalis* and *Helisoma trivolvis*, modulate the rate of embryonic development [13,17]. Both monoamines are also involved in the regulation of embryonic rotation within the egg capsule [18,19,20,21]. Thus, for a substantial period during the development of freshwater mollusks, monoaminergic neurons of the apical organ are the sole neural elements mediating the embryo’s response to external stimuli.

We hypothesized that the parentally-derived signal, which reflects the environmental condition, affects the differentiation pattern of embryonic apical neurons. Such plasticity in acquiring the final neurotransmitter phenotype by apical neurons is crucial for the realization of the adaptive behavioral responses of the embryo (rotation), which are fundamentally important for embryo growth and survival.

Experimental data on the modulation of serotonin levels in parental individuals confirm that an increase in 5-HT levels has long-term delayed effects on the development and behavior of *L. stagnalis* offspring. Specifically, offspring from parents with elevated 5-HT levels undergo metamorphosis more quickly, exhibit faster rotation within the egg capsule, and have better tolerance to hypoxia [22]. These findings suggest potential changes in the neurotransmitter composition of embryonic apical neurons, which may reflect the level of 5-HT in the maternal organism.

In our study, we demonstrate that the relative 5-HT level within apical organ neurons varies seasonally. These seasonal variations in 5-HT levels are likely to be crucial for embryonic fitness, as serotonin plays a key role in regulating embryo rotation within the egg and nutrient uptake during development [23]. Enhanced nutrient consumption could, in turn, lead to improved survival rates of the offspring and ultimately contribute to the overall reproductive success of the population. Experimental modulation of parental 5-HT levels, but not DA levels, altered the monoamine balance in apical neurons. While the relative DA levels in apical neurons remained unchanged, the relative 5-HT levels demonstrated a significant enhancement in the offspring of animals with elevated 5-HT levels in their gonads. This increase in the apical neurons’ relative 5-HT levels was accompanied by an enhanced expression of tryptophan hydroxylase (*TPH*), a key enzyme in serotonin synthesis. Notably, these parentally derived neurotransmitter alterations in the neurons of the progeny affected the locomotor activity of the embryos. It is generally believed that neurotransmitter specificity is determined during neuronal differentiation, and changes in the neurotransmitter phenotype, if they occur at all, are exceedingly rare. Such changes are typically induced by strong and direct stimuli such as hormones or growth factors [24], long-lasting osmotic stimulation [25], or under the functional insufficiency of the dopaminergic system [26]. Our findings provide the first experimental evidence of the targeted modification of the relative 5-HT/DA levels in the identified neurons, achieved through parental serotonin modulation, which reflects regular, conventional changes in the environmental conditions. By addressing the dynamic changes in neurotransmitter levels in the specific identified embryonic neurons, we aim to provide a more nuanced rationale for how these fluctuations could contribute to the adaptation of the developing individuals in response to environmental changes through parentally derived factors.

## 2. Results

### 2.1. Detection of Serotonin in Apical Neurons of Lymnaea stagnalis Veliger Stage Embryo

**Figure 1:**
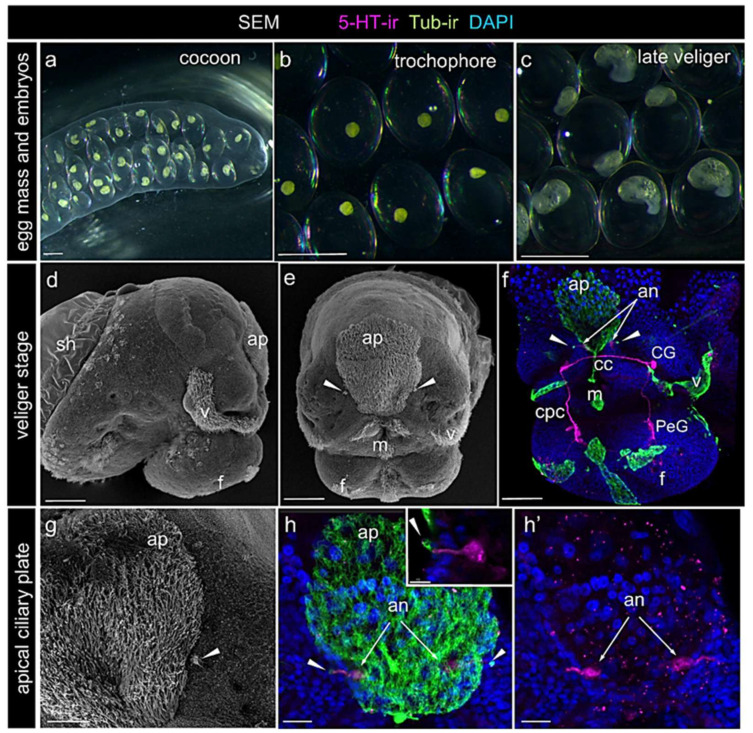
*Lymnaea stagnalis* embryos at the veliger stage. (**a**) Egg cocoon containing egg capsules. Image of live specimens captured using a stereomicroscope. (**b**,**c**) Egg capsules from the same egg mass at the trochophore and veliger stages, respectively. Images of live embryos captured using a stereomicroscope. Note the highly synchronized development of embryos within the same egg mass. (**d**,**e**) Embryo at the veliger stage, shown in right-side (**d**) and frontal (**e**) views, SEM images. Arrowheads indicate compact tufts of short cilia positioned symmetrically on either side of the ciliated apical plate. (**f**) Embryo at the veliger stage, frontal view, showing 5-HT-immunoreactive elements (5-HT-ir, magenta) and tubulin-immunoreactive ciliary structures (Tub-ir, green), DAPI marks the nuclei (dark blue). 5-HT-containing cells within forming ganglia, connectives, and commissures connecting ganglia rudiments are visible. Arrows indicate the positions of apical neurons beneath the ciliated apical plate. Note the presence of two small symmetrical ciliary tufts (arrowheads) on either side of the ciliary plate. (**g**) Close-up view of the ciliated apical plate and short ciliary tufts (arrowheads), SEM image. (**h**,**h’**,**h inset**) Close-up of the apical region showing 5-HT positive neurons (5-HT-ir, magenta) and tubulin immunoreactive ciliary structures (Tub-ir, green). Vase-shaped apical neurons (arrows) are clearly visible beneath the apical plate. (**h inset**) Thick apical processes extend to the surface, bearing a compact tuft of cilia (arrowheads). Abbreviations: an—apical neurons, ap—apical plate, cc—cerebral commissure, CG—cerebral ganglion, cpc—cerebro-pedal connective, f—foot, m—mouth, PeG—pedal ganglion, sh—shell, v—velum. Scale bars: (**a**–**c**) 1 mm, (**d**–**f**) 100 μm, (**g**–**h’**) 30 μm, and (**h inset**) 10 μm.

The *Lymnaea stagnalis* embryo develops inside egg capsules encased within a gelatinous cocoon (Figure 1a). Each egg capsule contains a single embryo, and all embryos develop highly synchronously within the same egg mass (Figure 1b,c). Approximately three days after oviposition, embryos reach the veliger stage. At this stage, the cranial–caudal axis of the embryo is longer than the dorsal–ventral axis, and the forming shell covers part of the visceral complex. The head and foot are distinctly separated. The ciliated velum is situated between the head and foot regions (Figure 1d). The ciliated apical plate is located medially on the head, above the mouth opening (Figure 1d,e). Two compact tufts of short cilia are visible on either side of the ciliated apical plate (Figure 1e,g).

We visualized the 5-HT-positive elements using antibodies against serotonin (5-HT-ir) and cilia using antibodies against acetylated alpha-tubulin (Tub-ir). 5-HT immunochemistry revealed neurons within the rudiments of the cerebral ganglia (CG) and pedal ganglia (PeG). The cerebral commissure (cc) connects the right and left CG and runs beneath the apical plate. Processes emanating from the ipsilateral ganglia form the cerebro-pedal connectives (cpc) (Figure 1f).

In some preparations, in addition to the developing neurons of CG and PeG, two 5-HT-positive cells were detected beneath the apical ciliary plate. The cell bodies were located symmetrically dorsal to the cerebral commissure (Figure 1f,h). Each cell had a vase-shaped body with a thick apical fiber bearing a tuft of short cilia (Figure 1h,h’,h inset). In our preparations, the intensity of 5-HT staining in the apical cells was weaker than that in the CG and PeG cells. Nonetheless, both the cell bodies and their apical processes were clearly visible (Figure 1f,h inset,h’).

The characteristic morphology, timing of appearance, and location enabled us to identify these cells as apical sensory neurons. These cells were previously described as DA-containing neurons belonging to the *L. stagnalis* apical sensory organ [16]. Additionally, they were occasionally observed to exhibit a low level of 5-HT-positive staining [13].

### 2.2. Serotonin Detection in L. stagnalis Apical Neurons Is Season-Dependent

**Figure 2:**
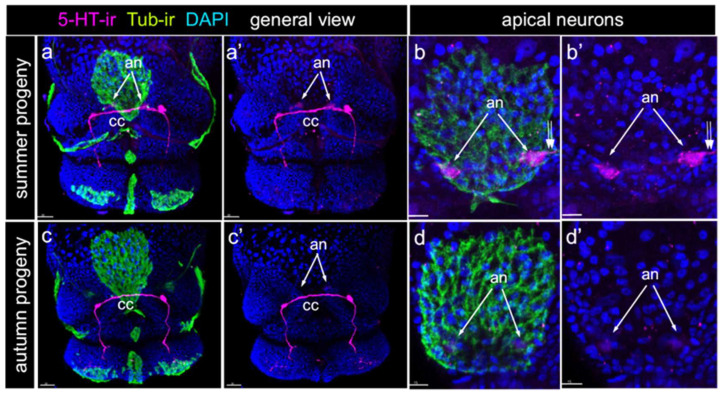
Expression of 5-HT-immunoreactivity in summer and autumn *Lymnaea stagnalis* progeny. 5-HT-immunoreactivity (5-HT-ir, magenta) and tub-immunoreactivity (Tub-ir, green) at the same veliger stage of different progeny, front view. DAPI marks nuclei (dark blue). (**a**–**b’**) Representative samples of preparations made in summer (June–July). 5-HT-ir visualizes the central ganglia rudiments, commissures, connectives, and apical neurons. Arrows indicate the position of apical neurons beneath the apical plate. (**a**,**a’**) General view; (**b**,**b’**) Close-up of the apical plate and apical neurons. Arrows indicate position of apical neurons; double arrows indicate the short sensory process of apical neuron. (**c**–**d’**) Similar regions of the same stage embryos but from preparations made in autumn (October–November). Arrows indicate the position where the faint outline of the apical neuron body is visible . (**c**,**c’**) General view; (**d**,**d’**) Close-up of the apical plate and apical neurons; arrows indicate position of apical neurons; no details of apical neuron morphology can be detected. Note the marked difference in the appearance of 5-HT-positive immunoreactivity in apical neurons between the summer and autumn veligers, while the central ganglia rudiments, connectives, and commissures demonstrated a similar appearance. Abbreviations: an—apical neurons, cc—cerebral commissure. Scale bars: (**a**,**a’**,**c**,**c’**) 30 μm, (**b**,**b’**,**d**,**d’**) 15 μm.

We analyzed the 5-HT-labeled preparations obtained during different seasons. Despite significant variations in the staining intensity across preparations, a distinct pattern emerged. A 5-HT-positive reaction within the apical neurons was consistently observed in preparations collected during summer. The staining pattern in these samples was uniform, with both cell bodies and processes clearly visible (Figure 2a–b’). In contrast, apical neurons were rarely detected in the preparations obtained during autumn. In the few instances where apical neurons were identified, the intensity of 5-HT staining was markedly lower compared with the summer preparations and was limited to the cell bodies, with no visible processes (Figure 2c–d’). Notably, other 5-HT-positive cells, connectives, and commissures were consistently visible across the preparations and exhibited a similar brightness regardless of the season (Figure 2a’,c’).

### 2.3. Pharmacological Modulation of Parental Serotonin Leads to Enhanced Relative 5-HT Level in Premature Oocytes and Follicle Cells Within the Gonad

The specifics of the preparation and storage prevented accurate comparisons of the brightness of the 5-HT-positive elements in the samples obtained during summer and autumn. However, previous studies have demonstrated that the critical difference between the physiological states of summer and autumn adult *L. stagnalis* is the serotonin level in the female reproductive system [22]. Based on this, we conducted pharmacological experiments to mimic the summer increase in the serotonin levels within the maternal reproductive system. Using a previously developed methodology for the pharmacological modulation of monoamine levels, we incubated mature snails in 5-HTP, the immediate biochemical precursor of 5-HT. In parallel, we incubated adult *L. stagnalis* in L-DOPA, the biochemical precursor of DA, to investigate whether similar changes would occur in catecholamine-containing elements in the female reproductive system. To assess changes in the 5-HT and DA levels, we measured the brightness of the labeled elements in the gonads and uterus. 5-HT was visualized using immunochemical detection, while DA was identified through the Faglu histochemical reaction.

**Figure 3:**
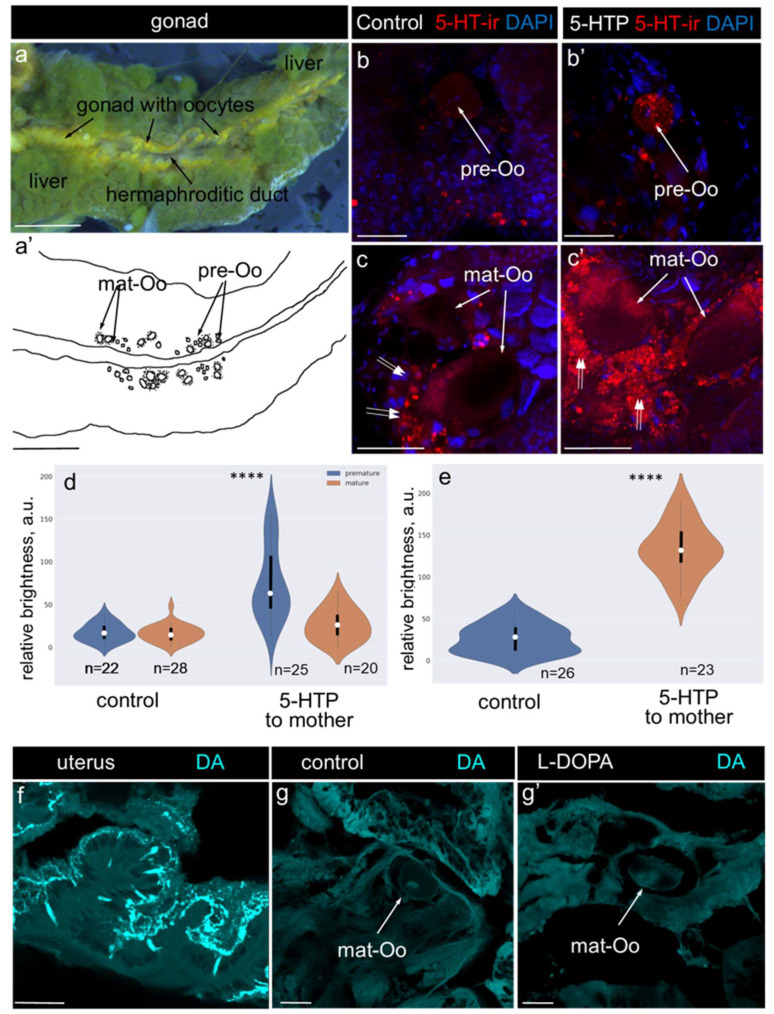
Changes in the serotonin and dopamine levels in the hermaphrodite glands of *L. stagnalis* after 5-HTP and L-DOPA treatment. (**a**,**a’**) Stereomicroscope photograph and schematic representation of the *L. stagnalis* hermaphroditic gland, respectively. The bright-yellow gonad with forming oocytes is surrounded by yellow-green liver tissue. Mature oocytes are surrounded by follicle cells. (**b**–**c’**) Immunochemical visualization of 5-HT (5-HT-ir, red) and nuclei (dark blue) in control and experimental gonad tissue. (**b**,**b’**) Premature oocytes (pre-Oo, arrows) in the gonads of the 5-HTP-treated (5-HTP) and control animals, respectively, stained with 5-HT antibodies. DAPI marks the nuclei. (**c**,**c’**) Mature oocytes (mat-Oo, arrows) surrounded by follicle cells (double arrows) in the gonads of the 5-HTP-treated (5-HTP) and control animals, respectively, stained with 5-HT antibodies (5-HT-ir, red). DAPI marks the nuclei (dark blue). (**d**,**e**) Violin plots illustrating the relative fluorescence brightness, correlating with the serotonin levels. (**d**) Relative brightness of the premature and mature oocytes in the control and 5-HTP-treated animals. Note the significant increase in the 5-HT levels in the premature oocytes of the 5-HTP-treated animals. (**g**) Relative brightness of the follicle cells in the control and 5-HTP-treated animals. 5-HT levels were significantly higher in the follicle cells of the 5-HTP-treated animals. In the violin plots, the mean value is represented by a dot, and quartiles are shown as bars. Asterisks mark statistical significance according to Wilcoxon rank-sum (Mann-Whitney) test, **** *p* < 0.0001. (**f**–**g’**) Histochemical visualization of DA (DA, cyan) in the female parts of the reproductive system. (**f**) DA-containing cells and their processes in the uterus part of a control animal. (**g**,**g’**) Oocytes in the gonads of both the control and L-DOPA-treated animals showed no positive histochemical reaction for DA. Abbreviations: mat-Oo—mature oocyte, pre-Oo—premature oocyte, double arrows marks follicle cells. Scale bars: (**a**,**a’**) 5 mm, (**b**,**b’**) 30 μm, (**c**,**c’**) 100 μm, (**f**–**g’**) 50 μm.

The gonad of *L. stagnalis*, containing developing oocytes, is surrounded by liver tissue. Oocytes of various sizes are typically observed within different lobes of the gonad. We classified oocytes smaller than 100 µm in diameter as premature, and those larger than 100 µm as mature. Additionally, mature oocytes are surrounded by small follicle cells (Figure 3a,a’).

After 5-HTP treatment, 5-HT immunoreactivity (5-HT-ir) appeared to be more pronounced in the premature oocytes and follicle cells, while it was less evident in the mature oocytes (Figure 3b–c’). Measurements of the relative fluorescence brightness in selected premature and mature oocytes (Figure 3d) and follicle cells (Figure 3e) confirmed a statistically significant increase in the 5-HT levels in 5-HTP-treated animals in the premature oocyte and follicle cells, respectively. To verify the presence of DA-containing cells in the female reproductive system, we used the uterus as a positive control. Brightly stained neuronal cell bodies and numerous processes were observed in the uterus (Figure 3f). However, no positive DA reaction was detected in the gonads of either the control or L-DOPA-treated animals (Figure 3g,g’).

Our results indicate that DA is not detectable in the oocytes or surrounding somatic cells following L-DOPA incubation. One possible explanation is that while 5-HT-positive gonadal cells might convert L-DOPA to DA, the oocytes themselves do not express the final enzyme required for DA synthesis—aromatic L-amino acid decarboxylase (AADC) [27]. It has been suggested that in mammalian gonads, surrounding follicle cells can synthesize and release 5-HT, which is then taken up by the oocytes [28]. A similar mechanism may operate in the gonads of *Lymnaea stagnalis*, as indicated by our findings (Figure 3b–c’). However, to date, there is no evidence of an active DA transporter in *L. stagnalis* oocytes, and our experimental data are in agreement with this assumption. Additionally, the lack of a DA signal in the oocytes and surrounding cells after L-DOPA incubation could be attributed to the limited sensitivity of the histochemical method for DA detection, especially in comparison with the immunohistochemical detection of 5-HT.

In summary, 5-HTP treatment enhanced the relative 5-HT levels in the premature oocytes and follicle cells, whereas L-DOPA treatment had no effect on the DA level in the oocytes.

### 2.4. Alteration in the 5-HT/DA Balance in the Apical Neurons of Offspring from Animals with Elevated 5-HT Levels

**Figure 4:**
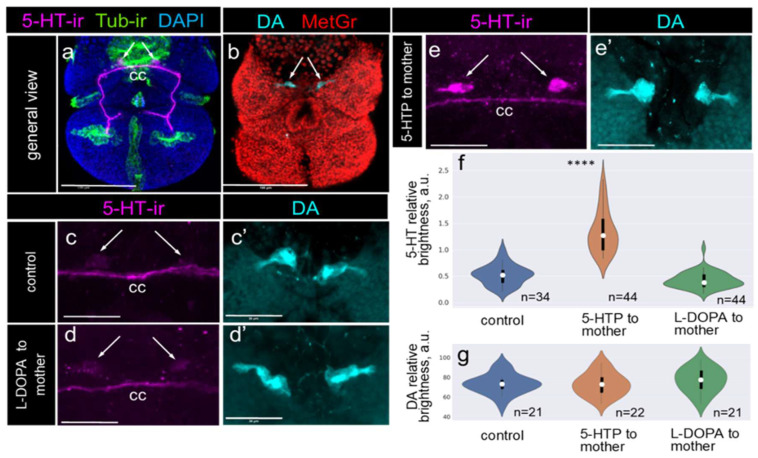
The 5-HT/DA balance in the apical neurons of the offspring reflects the parental 5-HT levels. 5-HT visualized via immunochemical labeling (5-HT-ir, magenta), DA detected using a histochemical reaction (DA, cyan), tubulin-immunoreactive ciliary structures (Tub-ir, green), and nuclei stained with DAPI (dark blue) and methylene green (MetGr, red). Arrows indicate apical neurons. (**a**) Visualization of 5-HT in a typical *L. stagnalis* veliger. (**b**) Visualization of DA in a typical *L. stagnalis* veliger. (**c**,**c’**) DA predominated over 5-HT in the apical neurons of the autumn *L. stagnalis* offspring. (**d**,**d’**) Both the 5-HT and DA levels remained unchanged in the offspring of L-DOPA-treated parents. (**e**,**e’**) Intensity of 5-HT-ir became more pronounced while DA staining remained unchanged in the apical neurons of offspring from the 5-HTP-treated parents. Note the consistent 5-HT-ir in the veliger cerebral commissure (cc) across all of the control and experimental groups, which was used for the normalization of the relative 5-HT level measurements. (**f**) Violin plots illustrating increased relative 5-HT levels in the apical neurons of offspring from the 5-HTP-treated parents. (**g**) Violin plots demonstrating stable relative DA levels in the apical neurons of offspring from the L-DOPA-treated parents. In the violin plots, the mean value is represented by a dot, and quartiles are shown as bars. Asterisks mark statistical significance according to Wilcoxon rank-sum (Mann-Whitney) test, **** *p* < 0.0001. Scale bars: (**a**,**b**) 100 μm, (**c**–**e’**) 30 μm.

Using a targeted approach to modulate the serotonin levels in the gonads of mature *Lymnaea stagnalis* (described above), we analyzed the changes in the relative 5-HT and DA levels in the apical neurons of their offspring. These experiments were conducted in autumn (October–December), a period when the 5-HT level in apical neurons is typically low, while DA is consistently present at the veliger stage (Figure 4a,b).

Apical neurons exhibited low 5-HT-ir intensity in both the control and L-DOPA-treated animals (Figure 4c,d). In contrast, the intensity of 5-HT-ir labeling was significantly elevated in the apical neurons of the 5-HTP-treated animals’ offspring (Figure 4e). Notably, at this developmental stage, the cerebral commissure had already formed and displayed a comparable intensity of 5-HT-ir expression across all experimental groups (Figure 4a–c). Thus, we used the intensity of 5-HT-ir in the cerebral commissure for normalized measurements of the relative 5-HT levels in apical neurons within the same embryo preparation. Along with the methodological approaches described in the Materials and Methods Section, these steps ensured that our measurements reflected the true relative intensity differences rather than the technical artifacts. Notably, DA, visualized through histochemical staining, showed consistent expression across all offspring groups (Figure 4c’,d’,e’).

The semi-quantitative analysis of fluorescence intensity in selected apical neurons confirmed a statistically significant increase in the relative 5-HT levels in the offspring of the 5-HTP-treated animals compared with the control offspring, while no such increase was observed in the offspring of the L-DOPA-treated animals (Figure 4f). Additionally, a comparative analysis of the relative DA level in apical neurons revealed no significant differences among the offspring of the control and experimental groups (Figure 4g).

These results demonstrate that the relative DA level in apical neurons remains stable regardless of the parental monoamine levels. In contrast, the relative 5-HT level was enhanced in apical neurons directly reflecting the parental 5-HT, but not DA, level. Together with the previous findings, this indicates that the 5-HT/DA balance in the apical neurons of the offspring specifically shifts to 5-HT in response to elevated parental 5-HT levels in the female reproductive system.

### 2.5. A Decrease in Maternal 5-HT or an Increase in 5-HT During the Embryo Cleavage Stage Does Not Affect the 5-HT/DA Relative Levels in Apical Neurons

To modulate the maternal 5-HT levels, we employed a pharmacological approach using chlorpromazine (CPZ) and the immediate serotonin biochemical precursor, 5-HTP. CPZ belongs to the class of typical antipsychotic drugs and functions by blocking receptors for several neurotransmitters, particularly those involved in the monoaminergic systems including dopamine, serotonin, norepinephrine, and histamine. Previous studies have shown that CPZ treatment reduced the 5-HT levels in the female reproductive system of adult *L. stagnalis*, while 5-HTP treatment during the cleavage stage of embryonic development increased the intracellular serotonin levels [22]. To determine whether these changes influenced the relative 5-HT level in the apical neurons of the offspring, we conducted two sets of experiments.

**Figure 5:**
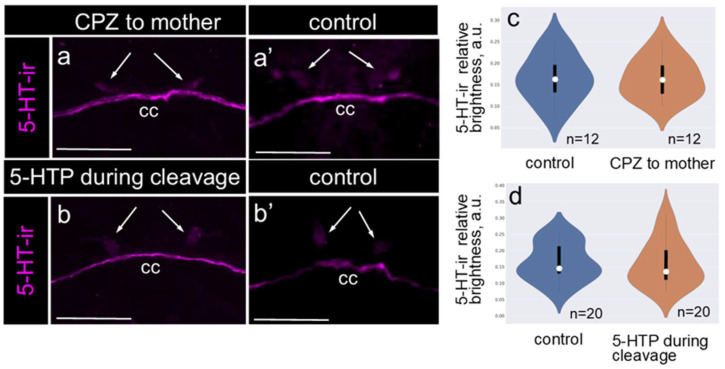
Relative 5-HT level in the apical neurons of the offspring from parents with reduced 5-HT levels (CPZ-treated) and embryos with increased intracellular 5-HT (5-HTP-treated) during the cleavage stage. 5-HT (5-HT-ir, magenta) was visualized using immunochemical labeling. Arrows indicate apical neurons. (**a**,**a’**) Visualization of 5-HT-ir in the offspring from the control and CPZ-treated animals, respectively. (**b**,**b’**) Visualization of 5-HT-ir in the control veligers and veligers developed from eggs incubated in 5-HTP during the cleavage stage. Note the consistent bright 5-HT-ir staining in the veliger cerebral commissure (cc) and the weak 5-HT-ir in apical neurons across all control and experimental groups. (**c**,**d**) Violin plots demonstrating stable relative 5-HT levels in the apical neurons of offspring from the CPZ-treated parents and embryos from eggs incubated in 5-HTP during the cleavage stage. In the violin plots, the mean value is represented by a dot, and quartiles are shown as bars. No statistical differences between groups were detected. Scale bars: (**a**–**b’**) 50 μm.

First, we compared the relative 5-HT level in the apical neurons of veligers from the control animals and those whose parents had been treated with CPZ (Figure 5a,a’). Second, we analyzed the relative 5-HT levels in the apical neurons of veligers from the control eggs and eggs treated with 5-HTP during the cleavage stage (Figure 5b,b’). In both cases, we observed no significant differences in the 5-HT-ir staining at the veliger stage. Intense 5-HT-ir was consistently detected in the cerebral commissure, and weak 5-HT-ir was observed in the apical neurons in both the control and experimental groups (Figure 5a–b’).

Semi-quantitative measurements of the fluorescence intensity confirmed that relative 5-HT levels in the apical neurons were unchanged in the offspring of the CPZ-treated parents compared with the controls (Figure 5c). Similarly, there was no difference in the relative 5-HT-ir intensity in the apical neurons of veligers from the control eggs versus eggs treated with 5-HTP during the cleavage stage (Figure 5d). In conclusion, neither a reduction in the parental 5-HT levels nor an increase in intracellular 5-HT during embryonic cleavage stages affects the relative 5-HT level of apical neurons in the offspring.

### 2.6. The Expression of the Tryptophan Hydroxylase Gene Is a Prerequisite for the Increased Levels of 5-HT in Apical Neurons

To determine the underlying molecular mechanisms responsible for increased serotonin levels in the apical neurons of embryos from the 5-HTP-treated parents, we analyzed the expression of tryptophan hydroxylase (*TPH*), a key enzyme involved in serotonin synthesis. *TPH* mRNA expression was analyzed across four developmental stages: blastula, gastrula, trochophore, and veliger. Additionally, we assessed the *TPH* expression in the central ganglia of both the control and experimental parents.

**Figure 6:**
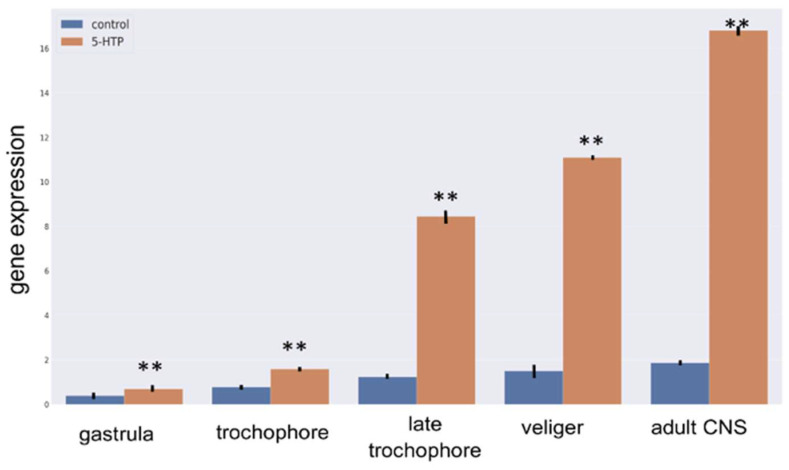
*TPH* expression profile in *L. stagnalis* embryos across developmental stages and in the adult central nervous system. *TPH* expression increased gradually according to the stage in both the control and experimental groups. Notably, progeny from parents with elevated serotonin levels exhibited significantly higher *TPH* gene expression compared with the controls. Asterisks mark statistical significance according to Wilcoxon rank-sum (Mann-Whitney) test, ** *p* < 0.01.

Although apical neurons do not show serotonin immunoreactivity until the late trochophore stage, *TPH* expression was detected as early as the gastrula stage. At all stages examined, the *TPH* expression levels were significantly higher in the progeny of parents with elevated serotonin levels (Figure 6). Moreover, increased *TPH* mRNA expression was observed in the central neurons of the 5-HTP-treated mature snails. Notably, the most substantial increase occurred during the late trochophore and veliger stages, coinciding with the onset of serotonin expression in the apical cells.

### 2.7. Embryo Rotation Within Eggs Responds to Changes in Parental Monoamine Level

**Figure 7:**
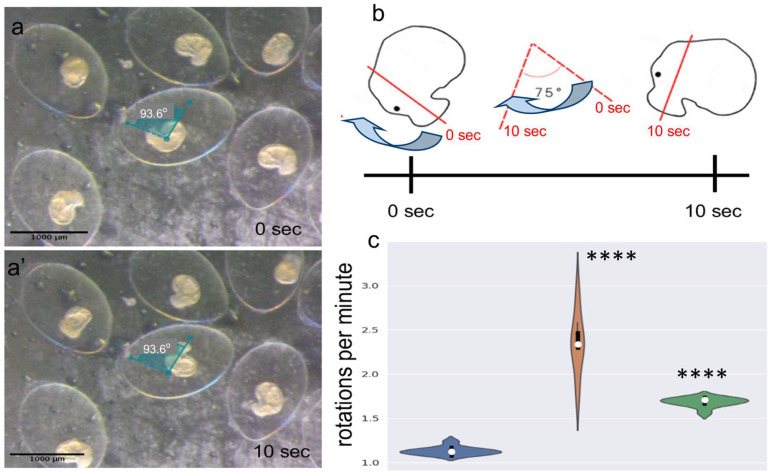
Representative pattern of embryo rotation and rotation speed in veligers from the 5-HTP- and L-DOPA-treated parents. (**a**,**a’**) Representative images of rotating embryos at time points 0 sec and 10 sec, respectively. The representative rotation angle for a selected embryo is indicated. (**b**) Schematic illustration of an embryo rotating over a 10-s interval (0 s and 10 s), demonstrating the method for measuring rotation speed. Arrows indicate the direction of rotation. The red line represents a virtual reference line drawn through the embryo’s upper head point and the center of the foot for angle quantification. (**c**) Violin plot showing the rotation speed of embryos from the control, 5-HTP-treated, and L-DOPA-treated snails. Asterisks mark statistical significance according to Wilcoxon rank-sum (Mann-Whitney) test, **** *p* < 0.0001. Scale bar: 1000 μm.

The characteristic behavior of embryos at the veliger stage is rotation within the egg capsule. We recorded videos of veliger-stage embryos and measured their rotation speed in rotations per minute (rpm) (Figure 7a,b). Analysis of the rotation speed showed an increase in embryos from both the 5-HTP-treated and L-DOPA-treated parents. In the control group, the average rotation speed was approximately 1.13 rpm. In contrast, embryos from the L-DOPA-treated parents rotated at an average of 1.68 rpm, while embryos from the 5-HTP-treated parents reached 2.35 rpm (Figure 7c).

Notably, the progeny of parents with elevated serotonin levels (5-HTP-treated) exhibited the highest rotation speed. This rotational behavior is critical for embryos, as it facilitates better oxygen and nutrient exchange, promotes optimal developmental rates, and supports overall embryo growth

## 3. Discussion

The findings of this study highlight the dynamic regulation of neurotransmitter phenotypes in *Lymnaea stagnalis* apical neurons and also contribute to the broader understanding of neuronal differentiation as a flexible process. Traditionally, neuronal differentiation has been viewed as a tightly controlled process that culminates in a stable neurotransmitter phenotype that corresponded to the functional role of a neuron. However, increasing evidence suggests that neurotransmitter expression can adapt in response to external or physiological stimuli, challenging the conventional notion of fixed neuronal identity [29]. By examining the dynamic changes in the neurotransmitter levels within specific, identified embryonic neurons, our findings offer a deeper understanding of how these fluctuations may contribute to the adaptation of developing individuals. These changes, mediated by parentally derived neurohumoral factors, may play a critical role in enabling offspring to respond to environmental challenges, ultimately enhancing their survival and the overall success of the population.

### 3.1. Maternal Influence as a Driver of Neuronal Plasticity

Our results demonstrate that increased maternal serotonin (5-HT) levels, which correspond to the respective seasonal environmental conditions, influence the serotonergic phenotype of embryonic apical neurons. This is consistent with findings in other systems, where maternal signals modulate offspring neurodevelopment.

For instance, in zebrafish, maternal glucocorticoids impact larval brain development, particularly primary neurogenesis in specific brain zones, resulting in enhanced boldness—an adaptive trait potentially advantageous in stressful environments [30]. Similarly, seasonal changes in maternal nutrients and melatonin levels affect the neurotransmitter pathways in the placental and fetal tissues of cows, illustrating the broader role of maternal signals in shaping offspring neurobiology [31]. In mice, maternal diet influences offspring brain development by modulating mTORC1 signaling via serotonin HTR6 receptors, impacting dendritic complexity and spine density, which serve as neuronal bases for memory formation [32]. Similarly, diet-dependent mTORC1 pathways are implicated in craniofacial shaping during embryogenesis in humans, mice, and zebrafish [33].

In *Lymnaea*, maternal serotonin appears to act as an epigenetic-like factor, conditioning embryonic neurons to express higher 5-HT levels in preparation for specific environmental conditions. Our previous studies demonstrated that the 5-HT levels in blastomeres influenced both embryonic development and juvenile behavior [22,34]. Increased serotonin at early cleavage stages accelerates development, enhances embryonic rotation, and leads to hatchlings that exhibit high activity, increased stress resilience, and greater fertility—traits associated with migratory behavior. In addition, we discovered that the *L. stagnalis* uterus contains a dense network of 5-HT neurons, thus the fertilized egg passing through the reproductive tract is exposed to 5-HT released by the parental neurons [35]. The 5-HT levels fluctuate due to external factors such as movement, stress, and seasonality, peaking in spring and summer and declining in autumn and winter [22,36,37,38]. Consequently, spring–summer offspring exhibit increased activity and migratory tendencies, while autumn–winter offspring are less active. We identified protein serotonylation as a key intracellular mechanism mediating the long-term effects of 5-HT. This process, in which serotonin covalently binds to proteins via transglutaminase (TGase), has been implicated in various physiological processes [39]. Notably, histone serotonylation has been linked to transcriptional regulation, providing strong evidence for the role of 5-HT in epigenetic signaling [40,41].

Our findings suggest a link between the seasonal maternal serotonin levels, embryonic neural differentiation, and behavioral traits. The ability of serotonin to induce long-term developmental changes through epigenetic mechanisms represents an adaptive strategy, aligning offspring neurobiology with anticipated environmental conditions. Pharmacological elevation of maternal 5-HT confirmed this connection, as it led to increased 5-HT levels in the oocytes and embryonic apical neurons, confirming the concept of maternal neurotransmitter-mediated programming.

### 3.2. Neurotransmitter Plasticity and Evolutionary Adaptation

The coexistence of serotonin and dopamine (DA) within apical neurons in *Lymnaea* embryos is a rare phenomenon, highlighting the plasticity of neuronal identity. Although most neurons express a single primary neurotransmitter, cases of dual-transmitter expression have been reported in specific regions of both invertebrate and vertebrate nervous systems. For instance, serotonergic-dopaminergic neurons have been observed in the lobster and molluscan nervous systems [2,3]. This dual expression may serve an integrative function, enabling neurons to process multiple signals and adapt their output based on changing conditions [42,43].

The alteration of neuronal phenotypes in response to specific conditions has also been documented in the adult mammalian brain. For instance, the expression of tyrosine hydroxylase (*TH*), the rate-limiting enzyme for DA synthesis, has been observed following the degeneration of dopaminergic neurons in the arcuate nucleus of adult rats [26]. Similarly, vasopressin-containing neurons in the rat supraoptic nucleus have been shown to synthesize *TH* in response to prolonged osmotic stimulation [25]. In both cases, the authors highlight that the induction of enzymes involved in monoamine synthesis in response to internal or external stimuli reflects the remarkable adaptive plasticity of neurons. Our findings add new data to the field of adaptive neuronal plasticity by demonstrating that apical neurons exhibit changes in neurotransmitter balance in response to environmental and maternal factors. Notably, the serotonergic profile of apical neurons increased significantly in the embryos of the 5-HTP-treated parents, while the DA levels remained stable. Such phenotypic plasticity likely represents an adaptive strategy, enabling embryos to optimize neural and behavioral responses to environmental fluctuations. Similar neurotransmitter switches have been observed in other species, such as the *Xenopus* tadpole, where environmental stimuli induce changes in neurotransmitter phenotype to optimize survival [44].

### 3.3. Mechanistic Insights into Neurotransmitter Regulation

The upregulation of tryptophan hydroxylase (*TPH*) expression in the embryos of the 5-HTP-treated parents provides a molecular explanation for the observed increase in serotonin levels. *TPH*, the rate-limiting enzyme in serotonin synthesis, is known to be dynamically regulated during development [45]. In our study, *TPH* expression began to increase at the gastrula stage, preceding the development of serotonergic neurons, and reached its peak during the trochophore-veliger stage. This developmental pattern aligns with previous observations that serotonergic systems in mollusks exhibit positive feedback regulation, where serotonin amplifies its own synthesis and release [46].

The feedback loop between maternal serotonin levels and embryonic neurotransmitter synthesis represents a unique form of mother-to-embryo signaling. Similar mechanisms of neurotransmitter feedback have been observed in a variety of invertebrates [47], suggesting that positive feedback regulation is a conserved feature of serotonergic systems.

### 3.4. Functional Implications of Neurotransmitter Changes

The changes in serotonin levels within apical neurons had functional consequences for embryonic behavior, specifically in increasing the rotation speed of veliger-stage embryos within their egg capsules. This behavior is critical for ensuring adequate oxygen and nutrient exchange during development [23]. Faster rotation rates in embryos of the 5-HTP-treated parents likely reflect an adaptive response to enhanced serotonergic activity, improving embryonic survival under hypoxic or nutrient-limited conditions [22].

Behavioral modulation by neurotransmitter plasticity has been documented across species. For example, dopamine levels influence larval swimming behavior in sea urchin larvae, contributing to dispersal and habitat selection [48]. Similarly, neurotransmitter adjustments in response to environmental cues have been shown to optimize physiological response to hypoxic stress in bivalve mollusks [49] and survival in other invertebrates, such as *Drosophila* larvae, where dopamine administration has a long-lasting effect on intestinal physiology and gut motility [50].

### 3.5. Evolutionary and Developmental Implications

The observed plasticity in neurotransmitter phenotype highlights the evolutionary significance of adaptive neuronal differentiation. Seasonal variation in maternal serotonin levels likely represents an evolutionary strategy to synchronize embryonic development with the environmental conditions, enhancing offspring survival. Such adjustments may have broader implications for understanding how environmental factors shape neural and behavioral phenotypes across generations.

Our findings also contribute to the growing evidence that neurotransmitter identity is not fixed but can be modified during development in response to external stimuli. While neurotransmitter switching is often associated with extreme environmental stress or hormonal signaling [51], our study demonstrates that subtle changes in parental physiology, such as increased serotonin levels, can also induce modifications in embryonic neurotransmitter phenotype.

## 4. Materials and Methods

### 4.1. Animals

The great pond snail, *Lymnaea stagnalis* L., is a freshwater aquatic pulmonate gastropod mollusk from the family Lymnaeidae. Laboratory cultures of *L. stagnalis* at the Institute of Developmental Biology RAS were originally donated by Vrije Universiteit, Amsterdam, and have been maintained under standard laboratory conditions since 1994. Adults are kept in aquariums at 22 ± 0.1 °C with changing filtered tap water, aeration, 16:8 light/dark cycles, and are fed lettuce ad libitum. These conditions have allowed the colony to be continuously renewed by obtaining embryos year-round. Mature snails produce one egg mass per day or one egg mass every 5–6 days, depending on the season. Spontaneously laid egg masses were collected daily between 9 a.m. and 11 a.m. and placed into 0.5 L aquaria. After hatching, young juveniles were transferred to 2 L aquaria, and once they reached maturity, they were moved to the stock colony.

The egg mass (cocoon) of *Lymnaea stagnalis* consists of 30–100 eggs, each containing a single embryo. Under normal conditions, the development of *L. stagnalis* is highly synchronous, with each developmental stage characterized by a specific set of morphological and behavioral features [22,52,53]. At 25 °C, the embryo reaches the gastrula stage within 24 h, the trochophore stage within 48 h, the late trochophore stage within 72 h, and the veliger stage within 80 h [52]. It is important to note that embryos within a single egg mass develop independently, and their responses to external stimuli may vary. Therefore, each embryo can be considered as an independent sample.

### 4.2. Pharmacological Manipulations of Monoamine Level in Parental Snail and Embryo

To manipulate the 5-HT levels in the parental *Lymnaea stagnalis*, we incubated mature snails with the respective 5-HT and DA precursors 5-hydroxy-L-tryptophan (5-HTP) and L-3,4-dihydroxyphenylalanine (L-DOPA) at a concentration of 0.1 mM each, and chlorpromazine (CZP) at a concentration of 1 μM. The 5-HTP, L-DOPA, and chlorpromazine hydrochloride were obtained from Sigma-Aldrich, St. Louis, MO, USA. All pharmacological solutions were freshly prepared in boiled, filtered water, with 50 µM ascorbic acid added to prevent oxidation. The same concentration of ascorbic acid was included in the water solutions for the control group. This incubation protocol with monoamine precursors has been shown to effectively increase the 5-HT and DA levels after 24 h of incubation, whereas incubation with CZP reduces the 5-HT levels in *L. stagnalis* tissues [13,22].

Experimental animals were transferred from the stock aquaria to 2-L tanks containing filtered aquarium water and maintained there for one month prior to the experiments to acclimate to the new culture conditions. For the experiments, the respective pharmacological solutions (5-HTP, L-DOPA, or CZP) were added to the tanks to achieve the working concentrations described above, along with ascorbic acid. Control animals were placed in water containing only ascorbic acid. Three tanks were used for each experimental group, with six mature snails per tank. The animals were fed lettuce ad libitum. After 24 h of incubation, the pharmacological solutions were replaced with filtered aquarium water. This water change typically induced egg-laying within 5–8 h. Egg masses were collected from each experimental group, and the eggs were removed from the egg mass and transferred to a 35 mm Petri dish with boiled, filtered water, and processed for further development. Embryos at the appropriate developmental stage (gastrula, trochophore, late trochophore, and veliger) were used for immunochemical visualization of the 5-HT-containing neurons, histochemical visualization of the DA-containing neurons, PCR-analysis, and embryo rotation assay.

**Figure 8:**
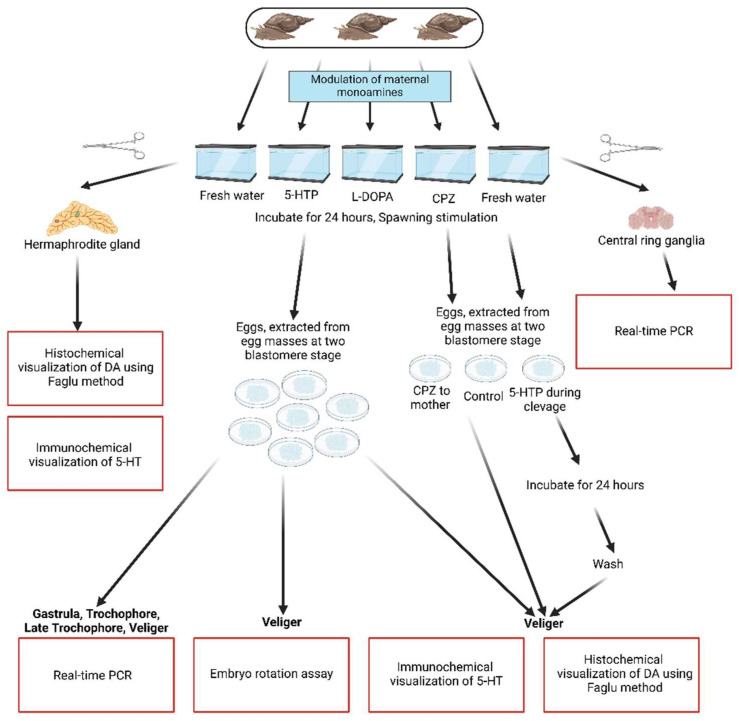
Schematic representation of the general experimental layout, procedures, and methodologies utilized in this study.

We also conducted experiments involving the pharmacological incubation of the embryos. Egg masses from the control parents were collected immediately after egg-laying. Eggs were carefully extracted from the jelly cocoons using forceps. Half of the eggs from a single egg mass were transferred to a 35 mm Petri dish containing 2 mL of 5-HTP (1 mM) with 0.1 mM ascorbic acid (experimental group), while the other half was placed in 0.1 mM ascorbic acid alone (control group). Embryos from each group were collected at the appropriate developmental stage and processed for further analysis. The experimental design is summarized in Figure 8.

The scheme illustrates the sequential steps and core methods employed throughout the experimental workflow. Key phases included: (1) the application of treatment protocols and control conditions to mature *Lymnaea stagnalis* aimed at modulating maternal monoamine levels; (2) collection of egg masses from different groups, extraction of eggs from the egg masses, and determination of the appropriate embryonic stage under a stereomicroscope; (3) extraction of embryos at appropriate stages from eggs for immunochemical and histochemical analyses as well as RT-PCR; and (4) quantification of the results obtained through the confocal microscopy imaging, RT-PCR analysis, and rotation assays.

Each stage is presented to highlight the integration of procedures, ensuring a comprehensive and systematic approach to addressing the research questions. The scheme serves as a visual summary for replicability and provides a contextual understanding of the experimental framework.

### 4.3. Immunochemical Visualization of 5-HT

To detect 5-HT and cilia in the developing *L. stagnalis* embryos and parts of the adult reproductive system, a standard immunocytochemical protocol [13] with minor modifications was employed.

Adult snails were anesthetized on ice, and the gonads and uteruses were dissected and immediately fixed in 4% paraformaldehyde (PFA) freshly prepared in 0.01 M phosphate-buffered saline (PBS, pH 7.4) overnight at 10 °C. The tissues were then thoroughly washed in PBS (several changes over 24 h at 10 °C) and transferred to 30% sucrose in PBS for 12 h at 10 °C. Frozen sections, 75 µm thick, were prepared using a Leica CM 1950 cryostat (Leica, Nussloch, Germany), mounted on slides, and processed for immunostaining.

Veliger-stage embryos were identified based on the standard developmental table for *L. stagnalis* [52] and selected using an SZ 40 Olympus stereomicroscope (Olympus, Tokyo, Japan). Eggs were carefully removed from jelly cocoons using forceps, and 15–20 eggs were placed on a microscope slide. Embryos were extracted from the eggs by gently squeezing them between two microscope slides and then washed through a 100 µm nylon mesh into a clean Petri dish containing 0.01 M PBS. The embryos were collected with a glass pipette, transferred to 2 mL Eppendorf tubes, and gently washed by pipetting. Samples were fixed in 4% PFA in PBS for 1 h at room temperature. After fixation, embryos were washed with 0.1% Triton X-100 (ThermoFisher, Waltham, MA, USA) in PBS (PBST) and then prepared for immunochemical staining. Before the antibody application, embryos were incubated for 2 h at room temperature in blocking solution containing 0.01 M PBS, 2.5% BSA, and 1% Triton X-100.

Samples were incubated overnight at 10 °C with the primary antibodies. For the gonads, a rabbit polyclonal anti-5-HT antibody (ImmunoStar, Hudson, WI , USA, #20080, dilution 1:2000) was used. For the embryos, a mixture of rabbit anti-5-HT antiserum and mouse monoclonal anti-acetylated α-tubulin antibody (Sigma-Aldrich, Munich, Germany, T-6793, dilution 1:4000) was applied. Following incubation, the samples were washed three times in PBST (10 min each) and then incubated for 12 h at 10 °C with secondary antibodies. For the gonads, goat anti-rabbit Alexa Fluor 555-conjugated IgG was used. For the embryos, Alexa Fluor 488 and goat anti-mouse Alexa Fluor 633-conjugated IgG (all from Molecular Probes, Eugene, OR, USA, diluted 1:1000 in PBST) were applied.

After incubation with the secondary antibodies, the samples were washed twice in 0.01 M PBS and transferred to 90% glycerol through a graded series (30%, 60%, and 90%). After 24 h of clarification at 10 °C, the gonad preparations were mounted in glass-bottom Petri dishes, and embryos were placed on microscope slides. The visceral part of each embryo was carefully removed using fine needles to orient them frontally. Importantly, the control and experimental embryos from a single experimental set were placed on the same slide to ensure equal image processing. Preparations were scanned using a confocal laser microscope, employing the appropriate excitation/emission settings.

As a control for staining specificity, the primary antibodies were omitted from the incubation solution. The specificity of the 5-HT and acetylated alpha-tubulin antibodies used for visualizing the 5-HT-containing neurons, fibers, and cilia has been demonstrated in previous studies on *Lymnaea stagnalis* [13,22].

### 4.4. Histochemical Visualization of DA Using the Faglu Method

To detect DA-containing cells, we employed the formaldehyde-glutaraldehyde (Faglu) method [54] with minor modifications. A solution containing 4% paraformaldehyde, 0.5% glutaraldehyde, and 30% sucrose in 0.01 M PBS (pH 7.4) (Faglu) was freshly prepared for the reaction.

Dissected adult gonads were placed in Faglu solution for 24 h at 10 °C. After incubation, 75 µm-thick frozen sections were prepared using a Leica CM 1950 cryostat (Leica, Germany). The slides with cryosections were left to dry in a dark, dry place for 24 h. Once dried, the samples were covered with mineral oil (Sigma-Aldrich, St. Louis, MO, USA) and sealed with a coverslip.

Veliger-stage embryos were extracted from the eggs as described above and incubated for 10 min in a 10 µM methyl green solution to visualize the nuclei. The embryos were then washed in PBS and transferred into freshly prepared Faglu solution for 24 h at 10 °C. After incubation, the embryos were placed on slides, and their visceral parts were carefully removed with fine needles to orient the samples frontally. Importantly, the control and experimental embryos from a single experimental set were placed on the same slide to ensure equal histochemical processing and image acquisition. Excess Faglu solution was thoroughly removed from the slides using a pipette with a narrow spout. The slides were then left to dry in a dark, dry place for 24 h. Once dried, the samples were covered with mineral oil (Sigma) and sealed with a coverslip.

The preparations were scanned using a confocal laser microscope with the appropriate excitation/emission settings for Faglu-induced DA fluorescence, according to the spectra described by Wreford and colleagues [55].

### 4.5. Image Acquisition

The confocal microscope’s capability to eliminate out-of-focus light enables optical sectioning through a specimen, allowing the fluorescence to be measured with exceptional spatial precision. Thus, despite some limitations, confocal images can be used for semi-quantitative measurements in cells and tissues. We carefully followed the guidance provided by Jonkman and colleagues [56] and Shihan and colleagues [57] to optimize the sample preparation, select an appropriate microscope, and configure the imaging parameters to obtain reliable semi-quantitative data.

Confocal images were acquired using Zeiss LSM880 (Carl Zeiss, Jena, Germany) and Leica TCS SP5 (Leica Microsystems, Nussloch, Germany) microscopes. Image processing and analysis were conducted using Zeiss software (ZEN Microscopy Software, version 3.2) and FIJI (http://fiji.sc/Fiji (accessed on 24 May 2023)). Scanning areas were selected to ensure all labeled objects of interest were included within the imaging volume. For each experimental series, standard settings—pinhole size, laser intensity, gain, and the number and thickness of the optical sections—were consistently applied to both the control and experimental samples. This standardized approach ensured the comparability of fluorescence intensity between the control and experimental samples.

### 4.6. Determining Fluorescence Intensity

Fluorescence intensity in the confocal microscopy images was used as a semi-quantitative measure to assess the relative differences in the presence of 5-HT and DA between experimental conditions. While not providing absolute quantification, this approach allows for meaningful comparisons of the monoamine relative levels revealed by the immunochemical (for 5-HT) and histochemical (for DA) staining.

For the semi-quantification of the relative 5-HT and DA levels, we used confocal imaging and fluorescence intensity measurements, following the method previously described [22]. To ensure that the fluorescence intensity measurements by confocal microscopy were used properly for semi-quantification, allowing for reliable relative comparisons under controlled conditions, we carefully followed the methodological guidelines established by Jonkman and colleagues [56] and Shihan and colleagues [57] in our study.

To ensure the reliability of our fluorescence measurements, we carefully controlled the sample preparation, imaging parameters, and data acquisition. Control and experimental embryos from the same experimental set were mounted on the same slide to ensure uniform immunostaining imaging and histochemical processing. Imaging was performed under identical settings for laser power, gain, and exposure time to maintain consistency across samples.

Fluorescence intensity was quantified using FIJI-ImageJ, following the established methodological guidelines [57]. Image analysis was performed on raw, unadjusted images to prevent bias. First, a Z-projection image was generated using the FIJI processing software (http://fiji.sc/Fiji, accessed on 24 May 2023) by selecting Image → Stacks → Z-stack. In the Analyze → Set Measurements menu, the “Mean gray value” and “Area” options were selected. The area of interest was then defined, such as the apical neurons and cerebral commissure in embryos, or specific cells in the hermaphroditic gonads, and the corresponding areas were processed for measurements. Next, the measurements of specific tissues of interest—apical neurons and oocytes—were normalized to adjacent tissues within the same preparation: the cerebral commissure for neurons and the area near the oocytes for oocytes, to enhance the measurement accuracy. These steps ensured that our measurements reflected the true relative intensity differences corresponding to the relative 5-HT and DA content rather than the technical artifacts. For oocytes in the adult gonads, the oocytes were initially classified into “premature” and “mature” categories based on size and location. To normalize the measurements in specific gonadal preparations, a background area near the cells was selected, measured, and used as the reference for normalization (normalized mean gray value = mean gray value of the cell − mean gray value of the background). For apical neurons, the fluorescence intensity was normalized to that of the cerebral commissure (normalized mean gray value of apical neuron = mean gray value of apical neuron/mean gray value of cerebral commissure). The normalized mean gray values were then used for statistical analysis. The normalized brightness is shown in each graph and is expressed in arbitrary units (brightness, a.u.).

### 4.7. Scanning Electron Microscopy

Embryos at the veliger stage were selected for scanning electron microscopy (SEM). A total of 20–30 specimens from three different egg masses were removed from the egg masses and eggs and transferred to 2 mL Eppendorf tubes containing 0.01 M PBS. After a quick wash through gentle pipetting, the samples were fixed in 2.5% glutaraldehyde in 0.01 M PBS, 24 h at 10 °C. After washing (3 times, 15 min each, 0.01 M PBS), the embryos were post-fixed in 1% OsO_4_ in 0.01 M PBS for 30 min. After post-fixation, the embryos washed in 0.01 M PBS (3 times, 15 min each) underwent dehydration in gradual ethanol solutions and were finally dehydrated in acetone.

For SEM, the embryos were dried in critical point, mounted on stubs using nail polish, and sputter-coated with Au-Pd. Samples were then analyzed using JEOL JSM 6380 (JEOL, Ltd., Tokyo, Japan), QuattroS (Thermo Fisher Scientific Inc., Waltham, MA, USA) and CamScan (Applied Beams, Beaverton, OR, USA) scanning electron microscopes at the Shared Research Facility ”Electron microscopy in life science” (Moscow State University, Moscow, Russia).

### 4.8. Real-Time PCR

Embryos at the gastrula, trochophore, late trochophore, and veliger stages (approximately 100 per sample) and adult nervous system rings (three) were placed into 1.5 mL Eppendorf tubes containing 0.5 mL of Tri Reagent (MRC, Cincinnati, OH, USA) and homogenized using syringes. RNA isolation and extraction were performed according to the manufacturer’s protocol. During the chloroform phase, glycogen (Biobagle, Saint Petersburg, Russia) was added to enhance RNA recovery.

The extracted RNA was used for cDNA synthesis in a final reaction volume of 20 µL, following the protocol and using reagents provided by Eurogen (Moscow, Russia). Each reaction mixture contained 2 µL of RNA solution and 2 µL of poly-T primer. The cDNA solutions were diluted to obtain a concentration of 1000 ng/µL, and DNA concentrations were measured using NanoDrop Eight (Thermo Scientific, Wilmington, DE, USA). Real-time PCR was performed using the SYBR-Green Low-ROX Mix (Eurogen, Russia). Each reaction mixture contained 1 µL of first-strand cDNA, 17 µL of water, 2 µL of primer mix, and 5 µL of SYBR reagent. PCR reactions were conducted on a QuantStudio 12K system (Life Technologies, Singapore) using 96-well plates. All amplifications were performed in three technical replicates. Gene expression levels were quantified using the comparative Ct method [58], with GAPDH serving as the reference gene for normalization.

Primers were designed using CloneManager software, version 10 and gene sequences obtained from the NCBI GenBank database (*GAPDH*—MH687363.1, *TPH*—AF129815.1). Nucleotide sequences of the primers used for real-time PCR are listed in Table 1.

### 4.9. Embryo Rotation Assay

Embryonic rotations at the veliger stage were monitored using a CCD camera (Zeiss AxioCam 208) attached to a stereomicroscope (Zeiss Stemi 305). To determine the dynamic rotation patterns, 3-minute videos were recorded using ZEN lite imaging software (https://www.zeiss.com/microscopy/en/products/software/zeiss-zen-lite.html (accessed on 12 June 2023). The angles of rotation were measured at 10-second intervals using FIJI software (http://fiji.sc/Fiji, accessed on 24 May 2023). The angular speed of embryo rotation was calculated and expressed as rotations per minute (rpm). Measurements were performed for a minimum of 10 embryos in each experimental group. A double-blind protocol was used for all of the measurements.

### 4.10. Statistics

Since none of our data passed the normal distribution test, we employed the Wilcoxon Signed Rank test for sample comparisons. Statistical analyses were performed according to guidelines established by Jonkman and colleagues [56]. Prism 6 (GraphPad, version 9.5.1) was used, differences were determined according to Wilcoxon rank-sum (Mann-Whitney) test and differences were considered statistically significant at *p* < 0.05. Violin plots (for graphs depicting the relative monoamine levels in the apical neurons and oocytes) and bar graphs (for graphs showing the embryonic rotation speed and *TPH* gene expression levels) were generated for all experimental groups.

All experiments were conducted with a minimum of three biological replicates, unless otherwise specified. For immunohistochemical and Faglu labeling of the gonads, a minimum of 15 locations from two gonads were analyzed per sample in each experimental series. Similarly, for immunohistochemical and Faglu labeling of the embryos, a minimum of 12 embryos were analyzed per sample in each experimental series.

## 5. Conclusions

In conclusion, this study underscores the importance of maternal influence and environmental factors in shaping neuronal differentiation and neurotransmitter plasticity. By linking maternal serotonin levels to changes in embryonic neurotransmitter composition and behavior, we provide new insights into the dynamic interplay between developmental programs and external signals. These findings have broad implications for understanding neuronal differentiation, behavioral plasticity, and the evolutionary strategies that optimize offspring survival.

## 6. Future Research and Possible Limitations

Our study underscores the critical role of maternal serotonin (5-HT) in shaping the neuronal phenotype and behavior of offspring. We presented a valuable model—the identified neurons of gastropod embryos—to investigate the molecular and genetic mechanisms underlying maternal 5-HT transfer and its regulation of neuronal differentiation. The neurons that adjust their 5-HT levels in response to maternal signals belong to the highly conserved sensory structure known as the apical organ. This structure, or its homologs, is present in the embryos of many invertebrate and vertebrate species. Comparative studies across diverse taxa are essential to determine whether this phenomenon is evolutionarily conserved or divergent, providing insights into its broader biological significance. Long-term research tracking the development, behavior, and reproductive success of offspring with altered 5-HT levels could further clarify the adaptive value of these maternal effects. Additionally, a deeper investigation into the molecular and genetic mechanisms governing maternal 5-HT transfer and its influence on neuronal phenotype is necessary. Identifying the specific signaling pathways, epigenetic modifications, and genetic factors involved will enhance our understanding of how environmental cues shape neuronal differentiation and behavioral adaptations.

While this study provides valuable insights into the role of maternal serotonin (5-HT) in shaping offspring neuronal phenotype and behavior, several limitations should be considered. First, the observed effects were restricted to apical organ neurons, with no detected impact on serotonergic neurons within the developing central ganglia. This limits the generalizability of our findings to broader populations of serotonergic neurons and should be verified. Future research should investigate whether maternal 5-HT influences other neuronal subpopulations including those within the central ganglia. Second, the pharmacological manipulations used to alter 5-HT levels, such as chlorpromazine and 5-HTP, may have had off-target effects that could have confounded the results. To refine our understanding, future studies should employ more specific genetic or molecular tools to precisely modulate the 5-HT levels and confirm the observed effects with higher specificity. Third, our study was conducted on *Lymnaea stagnalis*, a species whose embryos do not develop successfully outside the egg envelope, making them less suitable for advanced molecular genetic manipulations. Addressing this limitation by incorporating additional model organisms with established genetic toolkits will help broaden the conclusions to other taxa and neuronal subpopulations. Future research should aim to overcome these limitations to enhance our understanding of the interplay between maternal influence, environmental factors, and neurodevelopmental plasticity.

## Figures and Tables

**Table 1:** Nucleotide sequences of primers used for real-time PCR.

Primer Sequences	Gene Name
TAA CTG CTG CTG CTT CAC	*APDH*-F
GGA CTT CTT GGG AGA TAA CC	*GAPDH*-R
GCT CAC GCC CAC AGT AAA CAT C	*TPH*-F
GTC CAG CAA TGG TCA CAG TCT C	*TPH*-R

## Data Availability

The raw data supporting the conclusions of this article will be made available by the authors on request.
